# Detection of Colorectal Polyps from Colonoscopy Using Machine Learning: A Survey on Modern Techniques

**DOI:** 10.3390/s23031225

**Published:** 2023-01-20

**Authors:** Khaled ELKarazle, Valliappan Raman, Patrick Then, Caslon Chua

**Affiliations:** 1 School of Information and Communication Technologies, Swinburne University of Technology, Sarawak Campus, Kuching 93350, Malaysia; 2 Department of Artificial Intelligence and Data Science, Coimbatore Institute of Technology, Coimbatore 641014, India; 3 Department of Computer Science and Software Engineering, Swinburne University of Technology, Melbourne 3122, Australia

**Keywords:** automatic polyp detection, colorectal cancer, colorectal polyps, deep learning, computer vision

## Abstract

Given the increased interest in utilizing artificial intelligence as an assistive tool in the medical sector, colorectal polyp detection and classification using deep learning techniques has been an active area of research in recent years. The motivation for researching this topic is that physicians miss polyps from time to time due to fatigue and lack of experience carrying out the procedure. Unidentified polyps can cause further complications and ultimately lead to colorectal cancer (CRC), one of the leading causes of cancer mortality. Although various techniques have been presented recently, several key issues, such as the lack of enough training data, white light reflection, and blur affect the performance of such methods. This paper presents a survey on recently proposed methods for detecting polyps from colonoscopy. The survey covers benchmark dataset analysis, evaluation metrics, common challenges, standard methods of building polyp detectors and a review of the latest work in the literature. We conclude this paper by providing a precise analysis of the gaps and trends discovered in the reviewed literature for future work.

## 1. Introduction

Colorectal polyps are protrusions or bumps that develop in the colon as the body produces an excessive number of unwanted cells in the lining of the bowel. Although colorectal polyps are usually harmless at the initial stages, if left undetected and untreated over a long period, they could lead to colorectal cancer (CRC), one of the leading causes of cancer mortality [1,2,3]. According to the world health organization’s International Agency for Research on Cancer (IARC) [4], an estimated 2 million individuals were diagnosed with colorectal cancer in 2020 alone, with almost 1 million reported deaths due to the disease. The findings in [5,6] show that several common factors such as age, obesity, family history, excessive smoking and alcohol consumption and intestinal conditions like Crohn’s disease contribute to the development of colorectal polyps.

Colorectal polyps can form anywhere in the large intestine or rectum. However, most polyps are reported to form in the left part of the colon, known as the distal colon [5]. Some polyps form on the right side of the colon, known as the proximal colon, and are usually more challenging for colonoscopists to detect due to the shape of the colon [4,5]. 

Colorectal polyps come in various shapes and sizes; some are dangerous, while others are harmless bumps. Colorectal polyps are generally classified into several types, which can be either benign or malignant [1,2,3,4]. In Table 1, we present the various types of polyps.

Even though hyperplastic and tubular adenomas, which are benign, are the most common polyps found in patients, if they remain undetected and untreated over a long period, they can turn malignant [2,3].

To date, the standard method for detecting and diagnosing colorectal polyps has been through colonoscopy. Colonoscopy is a multistep process in which patients are first provided with a specific solution to consume as part of the bowel preparation phase. The bowel is prepared and cleaned to ensure that there are no obstructions, such as feces or other objects, that may affect the colonoscopy procedure. Next, the examination phase takes place, in which a colonoscopy that is equipped with light and a camera is inserted into the patient’s rectum [7].

From a machine learning perspective, colorectal polyp detection can be defined as the process of training a machine learning model to learn a set of features representing a polyp from a given image or live video stream. In some cases, those models are further fine-tuned, and a classification component is added to distinguish different classes of polyps.

Although there are multiple ways to build a colorectal polyp detection model, common preliminaries are usually followed to complete an end-to-end detection system. At first, a dataset consisting of either static photos or video frames is required to train the model. A typical dataset would be a combination of polyp images and either segmentation masks, bounding boxes coordinates, or both. Next, the samples are pre-processed and augmented using various techniques such as flips, random crops, and rotations. Augmentation is often needed because the current public datasets are small, and the samples do not cover all the possible scenarios, like variation in illuminations, the visual field and polyp sizes.

**Figure 1:**
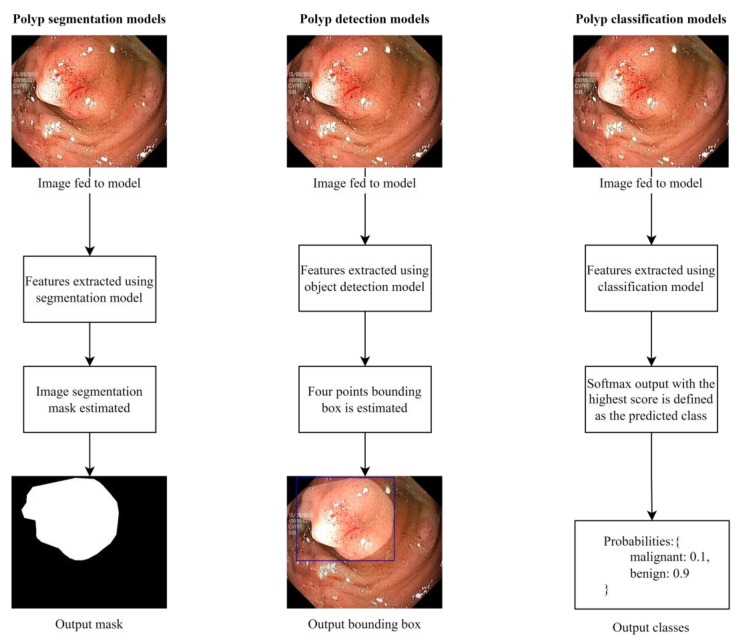
A comparison of the three common approaches to building colorectal polyp detectors. Colonoscopy images are taken from a public dataset known as Kvasir-SEG.

Colorectal polyp detection models are built with one of three objectives: (1) polyp segmentation, (2) polyp detection, and (3) polyp classification. Some models are trained to carry out multiple objectives simultaneously, such as detection or segmentation, followed by classification. When a model is trained to detect polyps, training images with their corresponding labels are fed to a network which learns how to localize a polyp in a given image. The label format used for detection is usually a combination of four coordinates representing the bounding box, a variable representing whether a polyp is present, and sometimes, the class of the polyp is added, provided that such information exists in the dataset. In contrast, segmentation models are trained to draw an image segmentation mask around a detected polyp. Segmentation models are trained on the colonoscopy images, and the corresponding image masks are used as the input labels. Finally, classification models are trained to classify polyps based on the group a polyp belongs to without identifying the location. In Figure 1, we illustrate the three different approaches.

**Figure 2:**
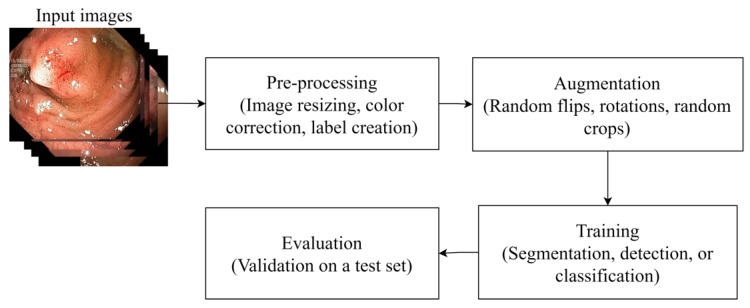
The standard steps required to build automatic polyp detection models. Samples in this illustration are taken from Kvasir-SEG.

Regardless of the desired approach, the pipeline of building an automated polyp detection system using machine learning is always identical, with the steps sample normalization, augmentation, model fine-tuning, and evaluation being the standard first steps. This process is summarized in Figure 2.

This article surveys the most recent trends and techniques for building automatic polyp detection models. The article covers various aspects, such as the existing public datasets available for training and testing, commonly reported challenges in the literature and the preferred deep learning architectures to build polyp detection models. Furthermore, we discuss and analyze the findings of the surveyed literature, and based on the analysis, we provide recommendations for future research.

The remainder of this paper is structured as follows: Section 2 presents our survey strategy and the criteria that make an article suitable for inclusion in our survey, Section 3 summarizes our contribution, Section 4 presents the challenges, Section 5 presents the datasets, Section 6 describes the standard architectures, Section 7 presents the performance metrics, Section 8 is a review of recently proposed work, Section 9 presents the discussion and analysis, and finally Section 10 concludes the paper. 

## 2. Survey Strategy

This survey focuses on the most recent methods and trends for detecting polyps using machine learning techniques. A total of 20 recently published articles were reviewed in this paper, and they were selected based on several conditions. First, we ensure that the reviewed article is relevant to the task of polyp detection by assessing the introduction, problem statement, proposed method and the reported result. Second, we look into the proposed method’s novelty and uniqueness and how it attempts to bridge some of the existing gaps. The third criterion we consider is the publication date, as we mainly focus on presenting the most current methods. Next, we look into the impact of the reviewed article based on the number of citations and mentions. In addition to the reviewed articles, our presentation of the challenges and common architectures is also based on an examination of the existing literature. During the literature analysis, we were interested in discovering the limitations that authors have reported in their work, which have been presented as existing challenges in this survey. Moreover, the polyp detection architectures reviewed in this survey are based on how frequently a specific design is mentioned in the reviewed work.

The articles were collected from journals from various databases, namely Google Scholar, ScienceDirect, the National Institute of Health (NIH) database, Nature, and SpringerLink. 

Some of the primary keywords used to search for relevant articles were “colorectal polyp detection”, “colorectal polyp segmentation”, and “colorectal polyp classification”. As for the datasets reviewed in this survey, we analyze the reviewed articles, and a list of standard datasets is made based on how many times a specific dataset is mentioned.

## 3. Contributions

Our contribution to the body of literature can be summarized in the following points:

1. We present a review of several recently proposed colorectal polyp detection methods.
2. We review and analyze the various architectures available to build colorectal polyp detectors.
3. Based on the current work, we present several common challenges researchers face when building colorectal polyp detection algorithms.
4. We present an analysis of the existing benchmark colorectal polyps datasets.
5. We analyze the findings in the reviewed literature and investigate the current gaps and trends.

## 4. Common Challenges

Building a colorectal polyp detection model to work on media fed from colonoscopies comes with several obstacles and challenges. Those challenges can be classified into intrinsic issues, i.e., limited to the detection model, and extrinsic issues, i.e., caused by other external factors. Some examples of intrinsic issues include the demand for high computational power to detect as many polyps as possible and overfitting caused by data disparity. In contrast, extrinsic issues include poor bowel preparation and reflection caused by the light emitted from the colonoscopy, which could confuse the model. In this section, we discuss some of the common challenges based on the current work of literature.

### 4.1. Data Disparity

The lack of enough diverse samples is a significant issue in most deep-learning applications. As polyps come in different shapes and sizes, the lack of enough diverse samples to cover most of the possible variations is a significant issue, as reported in [8,9]. This issue can be considered external as it is linked to the quality of the training samples. Although this issue can be addressed through various augmentation techniques or the introduction of artificially generated samples, misidentifying flat, depressed polyps still occur due to the lack of samples.

### 4.2. Poor Bowel Preparation

Proper bowel preparation is an essential preliminary step that, if done incorrectly, can increase misidentifications, as the model might identify non-polyp objects such as feces or normal blood vessels as polyps. Studies like [10,11] have reported that their methods misidentified polyp-like objects as being polyps, accounting for most of the detected false positives. Poor bowel preparation is an external factor that depends on the physician’s experience and the preparation process rather than fine-tuning the model or the introduction of new samples.

### 4.3. High Demand for Computational Resources

When working with live colonoscopy video streams, the efficiency of a model is a priority. However, optimizing the detection speed of a model usually means a drop in performance and an increase in inaccurate predictions. In [8,12], although promising performances were reported, the authors admitted that their proposed method demands sophisticated, high-end computational resources. The design of a model and the way it works is an internal problem that can be solved by bridging the gap between performance and speed.

### 4.4. Colonoscopy Light Reflection

As colonoscopies are equipped with light to ease navigation during the procedure, the reflection caused by the light source can affect the model performance during the detection phase. The reflection of white light can either hide the polyps or create a polyp-like anomaly in the colon. The researchers in [9,10] reported poor performance due to white light reflection. This issue is considered external, and can be solved through methods like the one introduced in [13].

### 4.5. Colonoscopy Viewpoints

The viewpoint of a colonoscopy refers to the field of vision of the camera attached to the colonoscope. In some cases, polyps may exist on the edges of a video frame, making them hard to detect even with the naked eye. In studies like [13,14], the researchers reported that one of the main challenges they faced was misidentifying actual polyps as non-polyp regions because the lesions were near the boundary of the video frame. Since this issue is related to the field of vision of the equipment itself, it can be classified as an external factor.

### 4.6. Pre-Training on Samples from an Irrelevant Domain

Transfer learning is considered the go-to solution in most deep-learning applications. The preference for using pre-trained models derives from the fact that such models are initially trained to handle much more complicated tasks, and the model’s hyperparameters are tweaked to solve a much simpler problem. In most of the existing polyp detection methods, transfer learning has been the preferred method for solving the problem; however, the original domain in which a model is pre-trained is primarily irrelevant to colorectal polyp detection. This discrepancy between colonoscopy images and the pre-training domain has resulted in poor performance, as reported in [15,16].

## 5. Benchmark Datasets

**Figure 3:**
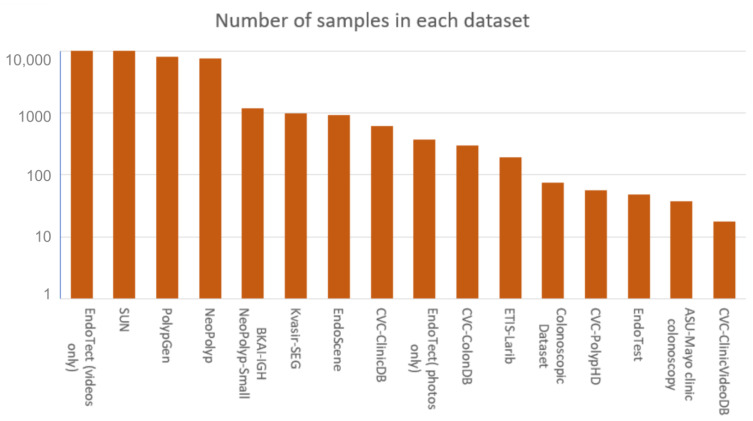
Total number of samples in every dataset.

Any computer vision problem requires a high-quality, diverse training dataset. The types of data available for colorectal polyp detectors include both live videos and static photos extracted from live video streams. This section analyzes the standard datasets used to build polyp detection models. In Table 2, a summary of all the datasets is presented and in Figure 3 a diagram describing the total number of samples for each dataset is illustrated.

### 5.1. Kvasir-SEG

The Kvasir-SEG dataset was introduced in 2020 by [17]. The Kvasir-SEG dataset consists of 1000 polyp images with their corresponding masks. The dataset samples were taken in unfiltered, real-life settings where the polyps were captured from different angles, and the samples are of different resolutions ranging between 332 × 487 to 1920 × 1072 pixels.

### 5.2. ETIS-Larib

ETIS-Larib was introduced in 2014 by [18], comprising 196 samples. Unlike Kvasir-SEG, the resolution of the samples in the ETIS-Larib dataset is fixed at 1225 × 996 pixels. The samples are all captured in unfiltered settings, and several images are blurry. The dataset is mainly used for testing due to the limited number of samples; however, some studies, such as [19,20], have used the ETIS-Larib dataset for training.

### 5.3. CVC-ClinicDB

CVC-ClinicDB was introduced by [21] in 2015, and is one of the most frequently used datasets. The dataset consists of 612 polyp static frames extracted from 31 colonoscopy sequences. The resolution of all the images is fixed at 384 × 288 pixels. The dataset has been widely used to test several segmentation methods, including the infamous U-NET [22].

### 5.4. CVC-ColonDB

CVC-ColonDB is another frequently used dataset introduced by [23] in 2012. There are 300 static samples in this dataset, and each image has a size of 574 × 500 pixels. The dataset was constructed based on 15 short colonoscopy video frames. The samples in this dataset are unique in terms of the polyps’ sizes, variations and types.

### 5.5. CVC-PolypHD

Introduced by [24] in 2017, CVC-PolypHD contains 56 high-resolution static samples, each sized at 1920 × 1080 pixels. Each sample is accompanied by a ground truth annotation mask that locates the position of the polyp. This dataset is not used for training as frequently as CVC-ColonDB, CVC-ClinciDB and Kvasir-SEG.

### 5.6. EndoTect

EndoTect was introduced as part of a challenge during the 25th International Conference on Pattern Recognition [25]. The dataset is relatively large, containing 110,079 static images and 373 videos of various resolutions. Out of the 110,079 images, 10,662 are labeled with the polyp classes, 99,417 images are not labeled, and 1000 samples are provided with their respective masks. The videos provided in this dataset amount to about 11.26 h and 1.1 million video frames.

### 5.7. BKAI-IGH NeoPolyp-Small

This dataset was proposed by [26] in 2022, and it is relatively medium in terms of size, with about 1200 static images. The dataset is already segregated, with 1000 samples detected for training and the remaining 200 samples for testing. The samples in this dataset are divided by class into neo-plastic and non-neoplastic, making it a suitable dataset for classification problems.

### 5.8. NeoPolyp

Neopolyp was introduced by [27] in 2020, and is an extension of BKAI-IGH NeoPolyp-Small. The dataset consists of 7500 labeled polyp images with fire-grained annotations. The images are of various resolutions and polyp sizes and shapes.

### 5.9. PolypGen

PolypGen [28,29,30] is a dataset used for segmentation and classification. Introduced in 2021, PolypGen was introduced as part of the 3rd International workshop and challenge on Endoscopic Computer Vision. The dataset consists of 8037 samples with 3762 positive samples (with polyps) and 4275 negative samples (without polyps). The samples are diverse in terms of resolution and quality.

### 5.10. EndoScene

EndoScene was introduced by [24] in 2017, and it is a result of merging two datasets, CVC-ColonDB and CVC-ClinicDB. The dataset consists of 912 samples, 300 from CVC-ColonDB and 612 from CVC-ClinicDB. The samples share the same characteristics as CVC-ColonDB and CVC-ClinicDB.

### 5.11. S.U.N. Colonoscopy Video Database

The Showa University and Nagoya University Database (SUN) was introduced by [31] in 2020. The dataset consists of 49,136 samples of 100 different polyps. In addition, the dataset contains 109,554 frames of non-polyp areas from live stream videos. The 100 polyps consist of the following: 82 Low-grade adenomas, seven Hyperplastic polyps, four Sessile serrated lesions, four High-grade adenomas, two traditional serrated adenomas and one invasive carcinoma. The polyps are of different shapes and sizes. The resolutions of the samples are diverse.

### 5.12. EndoTest

EndoTest was introduced by [32] in 2022, comprising two subsets. The first subset consists of 48 short videos with 22,856 manually annotated areas with polyps presence. Of the 22,856 samples, approximately 12,160 are positive, while the remaining 10,696 are negative. The second subset contains ten full-length colonoscopy videos with 230,898 annotated frames. Of the 230,989 frames, about 15% are positive samples, while the remaining are negative samples. The dataset is diverse in resolution and image quality.

### 5.13. ASU-Mayo Clinic Colonoscopy

ASU-Mayo Clinic is a video dataset introduced in 2017, and it is commonly used to build and test real-time polyp detection systems [33]. The dataset is divided into 38 video sequences and further segregated into 20 videos for training and 18 for testing. The training videos come with ground-truth masks. The resolution of the videos is not stated in the author’s work.

### 5.14. Colonoscopic Dataset

The colonoscopic dataset is another video dataset introduced in 2016 [34]. The dataset comprises 76 short colonoscopy videos, each sized 768 × 576 pixels and is mainly used in polyp classification problems. The polyps in this dataset are classified into three classes: (1) Hyperplastic, (2) Adenoma and (3) Serrated.

### 5.15. CVC-ClinicVideoDB

CVC-ClinicVideoDB was first introduced in 2017 by [35], and it is a video dataset that consists of 18 video sequences with a size of 768 × 576. Each video sample is accompanied by a polyp location mask which identifies the location of a polyp in a video. The videos cover a variety of properties, including different sizes, shapes, and variations of polyps.

## 6. Common Architectures

Building an automated polyp detection model depends primarily on the main objective of the model. In some instances, multiple architectures are combined to perform a particular task. For example, a model could be trained to detect and classify polyps; therefore, combining segmentation and classification into one model could be the ideal solution. This section presents the common architectures and designs available for carrying out polyp segmentation, classification and detection.

### 6.1. Segmentation Methods

Segmentation algorithms are deep-learning models commonly used to match the pixels of an image with a given class [36]. In colorectal polyp detection, segmentation algorithms are commonly used to locate polyps using a pre-defined mask during the training process.

#### 6.1.1. U-NET

U-Net was introduced in 2015, and since then, it has been one of the preferred segmentation networks in the medical imaging domain [22]. The network has an encoder, decoder and residual connections, and it consists of 23 convolutional layers, with each layer activated using the rectified linear unit (ReLU) function. U-NET has been used in relevant colorectal polyp detection studies [37,38].

#### 6.1.2. SegNet

SegNet is a semantic segmentation network that follows the encoder–decoder design and was introduced in 2015 [39]. Unlike U-Net, SegNet does not have a series of residual connections; however, it follows the encoder–decoder design and a pixel-wise classification layer. The encoder consists of 13 convolutional layers identical to the VGG16 design [40], and it downsamples the input image. The decoder consists of 13 convolutional layers; however, the pooling layers are replaced by upsampling layers to upsample the input image. Studies such as [41] have utilized SegNet to segment colonoscopy images.

#### 6.1.3. Fully Convolutional Networks (FCN)

The fully convolutional network (FCN) is a segmentation network that comprises convolutional layers only [42]. According to the authors, FCN is flexible, since it does not require a specific input size, and the training speed is much faster because there are no dense layers in the network. The network has a downsampling path, upsampling path and several skip connections to recover any lost information during the downsampling process. In [43,44], the authors used FCNs to detect colorectal polyps during colonoscopy.

#### 6.1.4. Pyramid Scene Parsing Network (PSPNet)

Pyramid Scene Parsing Network (PSPNet) was introduced in 2017 by [45]. The core concept behind the network’s design is that it uses a pyramid parsing module that produces more accurate global context information using different-region context aggregation. PSNet depends on a pre-trained convolutional neural network and dilated convolution to obtain a feature map. PSPNet usage in colorectal polyp detection is not as common as SegNet and U-Net; however, a recent study by [46] used the network and presented a comparison of its performance with F.C.N., SegNet and U-Net.

### 6.2. Object Detection Methods

Object detection algorithms such as YOLO and R-CNN networks are commonly used to detect specific objects of interest from medical images, including colorectal polyps. A typical object detection algorithm would draw a bounding box around a detected object in a given scene [47].

#### 6.2.1. Region-Based Convolutional Neural Networks (RCNN)

Region-based CNN is an object detection algorithm introduced in 2014 by [48]. R-CNN uses high-capacity convolutional neural networks to localize objects of interest and independently extracts features from each region of interest for further processing. Several studies, such as [49,50,51], have proven that RCNN-based polyp detectors can effectively locate polyps of different shapes and sizes.

#### 6.2.2. Faster Region-Based Convolutional Neural Networks (Faster R-CNN)

A faster version of RCNN was introduced by [52] in 2015, known as Faster R-CNN. This algorithm is similar to the original RCNN; however, it uses a region proposal network (RPN) that shares all the convolutional features with the detection network, making it an appropriate solution for real-time detection. Fast R-CNN was used to build several real-time polyp detection models, such as [53,54,55].

#### 6.2.3. Single-Shot Detector (SSD)

Single-shot detector (SSD) was introduced in 2016 by [56], and it depends on a single deep neural network to detect an object. The SSD model discretizes the output space of bounding boxes into several boxes with different aspect ratios. After the bounding boxes are discretized, the network scales per feature map location. The location of an object of interest is predicted using several feature maps with different resolutions. SSD detectors were used in recently proposed colorectal polyp detection methods such as [57,58].

#### 6.2.4. You Only Look Once (YOLO)

The first version of You Only Look Once (YOLO) was introduced in 2016 by [59], and it is one of the more popular object detection algorithms. The original algorithm, YOLOv1, treats detection as a regression problem in which a single neural network predicts bounding boxes and class probabilities from an entire image in one iteration. The YOLO architecture has been the preferred go-to solution for real-time detection tasks, as it can process 45 frames per second. Over the years, the YOLO algorithm has been modified, and better versions such as YOLOv2, YOLOv3, YOLOv4 and YOLOv5 have been introduced. The current work [60,61,62,63] shows that the YOLO algorithm has been used more frequently than other detection algorithms.

### 6.3. Pre-Trained Convolutional Neural Network Models

A pre-trained network is a model trained on a much larger dataset to solve a complex problem and then fine-tuned to work on a more straightforward task. This process of using existing knowledge to solve another problem is known as transfer learning. Several polyp classification methods have utilized pre-trained models as it is much faster to fine-tune and more accurate than classification methods built from scratch [64,65,66].

#### 6.3.1. VGG16

The VGG16 network was introduced by [40], and it is a standard network used in many computer vision tasks, including colorectal polyp classification. The network consists of 16 convolutional layers and takes an input size of 224 × 224 pixels. The network was trained on a large dataset known as ImageNet, a collection of 14 million images belonging to 22,000 classes [67]. The methods proposed in both [68,69] used the VGG16 model to extract polyp features from colonoscopy images.

#### 6.3.2. VGG19

VGG19 is identical to the VGG16 network except for the number of layers and model size. VGG19 has 19 convolutional layers and accepts an input size of 224 × 224, similar to VGG16. The VGG19 was trained on the ImageNet dataset as well. The authors in [70,71] used VGG19 as the backbone feature extraction network to automatically classify polyp images.

#### 6.3.3. ResNet50

ResNet50 is another pre-trained network used widely in various computer vision tasks, requiring an input size of 227 × 227 pixels [72]. ResNet50 is 50 layers deep with 48 convolutional layers, one max-pooling layer, and a single average-pooling layer. ResNet50 implements skip connections to allow information to flow directly without passing through an activation function. ResNet50 was trained on the ImageNet dataset and can classify 1000 categories. ResNet50 was the backbone network to detect and segment colorectal polyps in studies such as [73,74].

#### 6.3.4. Xception

Xception consists of 71 depthwise-separable convolutional layers [75]. The network takes an input size of 299 × 299 pixels and was trained on ImageNet. Xception is not used as frequently as the VGG networks or ResNet; however, [76] attempted to analyze the classification performance of the Xception model with the Swish activation function on colorectal polyps.

#### 6.3.5. AlexNet

AlexNet is designed as a typical convolutional neural network with pooling and dense layers [77]. AlexNet is only eight layers deep and takes an input size of 227 × 227, and it was trained on the ImageNet dataset to classify 1000 different objects. Although AlexNet is not the most common go-to network for classifying polyps, several recent studies have reported that AlexNet was the best-performing classification model [78,79,80].

#### 6.3.6. GoogLeNet

GoogLeNet is a convolutional neural network with 22 layers [81]. The network takes images of size 224 × 224 by default. Similarly to the other network covered in this section, it was trained on the ImageNet dataset to classify more than 1000 images. GoogLeNet was used to identify and localize polyps in [82,83,84], but it consistently underperformed when used as a standalone network. Therefore, GoogLeNet is mainly used as part of ensemble-based architectures.

## 7. Performance Evaluation

Evaluating the performance of any deep learning application is a critical step, as it provides an image of how generalized a model is. For automated colorectal polyp detectors, various metrics can be used to evaluate a model’s performance. The available metrics are used in specific scenarios, depending on whether the task involves segmentation, localization or classification.

### 7.1. Classification and Localization Metrics

The most common combination of metrics used to evaluate automatic polyp detectors and classifiers is the precision, recall and F1 scores. Precision is used to calculate the number of correctly predicted outputs over the total number of correct and incorrect predictions, and it can be represented as follows:

(1) $$\mathrm{Precision}=\frac{\mathrm{TP}}{\mathrm{TP}+\mathrm{FP}}$$

TP is the number of true positives, and FP represents the number of false positives. The recall metric is another measurement used to assess the model’s ability to correctly detect positive samples. The recall formula is defined as follows:

(2) $$\mathrm{Recall}=\frac{\mathrm{TP}}{\mathrm{TP}+\mathrm{FN}}$$

In the equation above, TP remains the number of true positives, and FN is the number of false negatives. When both precision and recall formulae are combined, the F1 measurement can be obtained using the following formula:

(3) $$F1=2\frac{\mathrm{Precision}\;\times \;\mathrm{Recall}}{\mathrm{Precision}+\mathrm{Recall}}$$

The main objective when using the abovementioned formulae is to achieve a value that is either 1 or close to 1, which indicates that the model performs well. The three formulae are usually used to evaluate how well a model can classify or detect polyps in a given image.

Another standard set of metrics among researchers is sensitivity and specificity measures. Sensitivity simply measures how well a model can detect positive instances. In contrast, specificity measures how well a model can identify true negatives. Both measurements can be represented as follows:

(4) $$\mathrm{Sensitivity}=\frac{\mathrm{TP}}{\mathrm{TP}+\mathrm{FN}}$$

(5) $$\mathrm{Specificity}\;=\frac{\mathrm{TN}}{\mathrm{TN}+\mathrm{FP}}$$

Accuracy is another metric used to evaluate polyp detection methods; however, it is unreliable when the dataset is unbalanced; therefore, researchers prefer using the F1 score to assess performance. Accuracy is defined as follows:

(6) $$\mathrm{Accuracy}=\frac{\mathrm{TP}+\mathrm{TN}}{\mathrm{TP}+\mathrm{TN}+\mathrm{FP}+\mathrm{FN}}$$

TP is the number of true positives, TN is the number of true negatives, FP is the number of false positives, and FN is the number of false negatives. A preferable metric that can be used to assess polyp detection models built using YOLO, RCNN and Fast-RCNN architectures is mean average precision (mAP). Mean average precision is a better metric for evaluating object detection algorithms, as it uses both recall and precision to assess a model. The mAP metric can be represented using the following formula:

(7) $$\mathrm{mAP}=\frac{1}{n}\overset{k=n}{\underset{k=1}{\sum }}{\mathrm{AP}}_{k}$$

$AP_{k}$ represents the average precision of class k, while n is the number of classes

### 7.2. Segmentation Metrics

The standard evaluation metric for polyp segmentation tasks is the Intersection-Over-Union (IoU), also known as the Jaccard Index. The IoU formula is defined as follows:

(8) $$\mathrm{IoU}\left(A,B\right)=\frac{\left|A\;\cap B\right|}{\left|A\;\cup B\right|}$$

In Equation (8), |A ∩B| is the area of overlap and |A ∪B| is the area of union. An IoU that is greater than or equal to 0.5 is considered acceptable.

## 8. Survey of Existing Work

This section presents a survey on recently proposed colorectal polyp detection methods. The survey covers the details of each method, datasets, reported performance, and any reported limitations.

In [8], the authors introduced a real-time polyp detection algorithm based on modified YOLOv3 and YOLOv4 algorithms. The authors modified the YOLOv3 network by replacing the original DarkNet53 with a CSPNet [85]. As for the YOLOv4 network, a modified CSPNet denoted as CSPDarkNet53 was introduced as the new backbone of the YOLOv4 network. In addition, another modification was made to the YOLO networks, in which SiLU replaced all the ReLU activation functions. To overcome overfitting, the authors utilized various augmentation methods such as rotation, flip, scale, and translate. The main objective of this study was to improve the performance of both YOLO networks by modifying their core structure. The proposed method was trained on the SUN and PICCOLO Widefield datasets and scored a precision of 90.61, recall of 91.04 and F1 score of 90.82 on the Etis-Larib dataset. Some of the main limitations reported in this study include an increase in misclassification when the model encountered fecal matter and bubbles.

In another study, a saliency detection network was introduced to detect polyps from static polyp images [13]. In their study, the authors used Neutrosophic theory to decrease the effect of white light reflections caused by the colonoscopy light. The authors introduced an image-suppressing technique to rebuild a colonoscopy image without the white light reflection using a single-value Neutrosophic set (SVNS). The specular regions are then recovered using a dynamic window that searches non-specular pixels near each specular pixel. An 8 × 8 window is used with a specific size and rotated counter-clockwise until the whole image is covered and all specular regions are recovered. The RGB pixels’ average value is used to paint the specular pixels in the recovered image. The authors introduced a saliency network known as NeutSS-PLS, inspired by the design of U-Net and DSS, for the detection and segmentation part. The introduced network had two-level short connections on both sides of the VGG. The training was conducted on the EndoScene and Kvasir-SEG datasets, and precision and F1 scores of 92.30 and 92.40 were reported, respectively. The proposed method struggled to identify polyps near the boundary of colonoscopy images.

In [10], an automatic polyp detection and segmentation system was introduced. The proposed method is called shuffle-efficient channel attention network (sECA-NET), and it segments colonoscopy images to detect polyps. At first, a CNN is applied to extract the feature map from an input image and a region proposal network (RPN), which predicts bounding boxes around polyps in the feature map, is developed. A region of interest align (RoiAlign) is applied to extract features from the feature map based on the bounding box of each detected object. Two parallel branches then compute the extracted features for every ROI. In the first branch, the features are computed by the fully connected layers, followed by softmax activation, and then a bounding box regression is performed. The second branch concerns mask segmentation, which predicts the category of each pixel in the region of interest. The proposed method was trained on the CVC-ClinicDB, ETIS-Larib and Kvasir-SEG. A private cross-validation dataset was used to evaluate the proposed idea. The authors reported a precision score of 94.9%, a recall score of 96.9% and an F1 score of 95.9%.

A dual-path CNN architecture was proposed in a different study by [9]. The proposed model takes an input colonoscopy image and produces a label corresponding to one of two classes: polyp and non-polyp. The first step of this method is image enhancement, in which the images are transformed into HSV color space. The V value in the HSV space is extracted using a multiscale Gaussian function; then, gamma correction is executed to correct the image’s brightness. After conversion, image fusion is conducted, in which the HSV image is converted back to RGB. Next, a custom, dual-path CNN that is eight layers deep extracts features from the processed image; then, the features are fed to a sigmoid layer, and mapped onto polyp and non-polyp. The training of this network was done on the CVC-ClinicDB, while testing was conducted on the CVC-ColonDB and ETIS-Larib datasets. The model performed best on CVC-ColonDB, with a precision of 100%, recall of 99.20% and F1 score of 99.60%. The main limitation observed with this method is that the model assumes the location of the polyp manually, which is impractical when working with real-life samples.

Inspired by U-NET, the authors of [14] introduced Y-Net to detect polyps in colonoscopy images. The proposed Y-Net consists of two encoders and a single decoder, and it can be trained on a limited number of samples. Both encoders follow the VGG19 design, while the decoder is a custom-built CNN with five deconvolutional blocks and one final convolution block. The first encoder is initialized on the ImageNet weights, the second encoder is initialized using the Xavier normal initializer, and both use the SELU activation function instead of ReLU. Y-Net was trained and tested on the ASU-Mayo dataset without a cross-validation dataset. The authors reported a precision of 87.4, recall of 84.4% and F1 scores of 85.9%. The authors reported that this method did not work well with reflections, polyp-shaped objects, and flat lesions.

The authors in [58] proposed a deep learning-based method to detect and classify polyps from colonoscopy images. The proposed method utilizes the single-shot detector algorithm (SSD) to locate a polyp in a given image. At first, the images are manually annotated by specialists in which a bounding box is drawn around each polyp, and this bounding box is used to train the proposed model algorithm. After annotation, the images are pre-processed using dynamic histogram equalization to enhance the quality of the input image. The authors used a dataset provided by the University of Leeds, and an accuracy of 92% was reported. This method was not evaluated against any available datasets.

A method that utilizes ensemble learning was proposed in [83], in which three models were used together to classify whether a polyp exists in a given image. The authors in this method stacked three pre-trained classifiers: (1) ResNet101, (2) GoogLeNet, and (3) Xception. The proposed model attempts to first classify whether a polyp exists in an image; the model then classifies the polyp again to check whether the detected lesion is malignant or benign. The three models extract features independently then a weighted majority voting representing whether a polyp exists and whether it is malignant or benign is produced. The training and testing were performed using a private collection of colonoscopy images and the Kvasir-SEG datasets. The authors reported precision and recall scores of 98.6 and 98.01, respectively, for the polyp detection task. In addition, precision and recall of 98.66 and 96.73 were reported for the malignant/benign classification task.

In a different approach, researchers in [11] used a no-code, deep-learning platform to predict colorectal polyps from colonoscopy images. The authors used a platform known as Neuro-T, as it had the most user-friendly GUI and it produced the best performance scores. The authors fed the system with white light colonoscopy images that were manually labeled with ground-truth labels according to a pathological evaluation. The authors acquired a different colonoscopy dataset to evaluate the proposed method. The authors reported a precision of 78.5, a recall of 78.8 and an F1 score of 78.6. One of the main limitations reported in this paper is that the system consistently misclassified normal blood vessels as polyps.

In [86], the authors attempt to combine the SWIN [87] transformer and EfficientNet [88] to segment and detect polyps from colonoscopy images. The proposed method combines architectures to capture all the critical global information through the SWIN transformer and all the local features using the EfficientNet. The proposed method has a multi-dilation convolutional block to refine all the local features extracted by EfficientNet and the SWIN transformer separately. In addition, a multi-feature aggregation block is added to aggregate both the global and local features. Once the features are refined and aggregated, an attentive block receives the features, and a polyp mask is built. The proposed method was trained on the Kvasir-SEG and CVC-ClinicDB datasets and tested on the CVC-ColonDB, ETIS-Larib and Endoscene datasets. During the evaluation stage, the authors reported a mean dice coefficient of 0.906, an IoU of 0.842, a mean weighted F-measure of 0.88, mean absolute error of 0.001.

In [89], the authors introduced a deep learning-based method to detect colorectal polyps from colonoscopy images. The proposed method can identify high-risk regions and classify polyps in a given region using a traditional machine-learning algorithm. For polyp detection, the authors use the Faster-RCNN network combined with a ResNet101 to extract the features of the detected polyp. A gradient-boosted decision tree classifier takes the output of Faster-RCNN and predicts whether a given region is high or low risk. The authors used a dataset provided by Singapore General Hospital; no cross-validation on a different dataset was mentioned in this work. The authors reported a sensitivity of 97.4%, specificity of 60.3%, AUC of 91.7% and F1 score of 97.4%.

In a study by [90], the authors introduced a deep learning method to differentiate premalignant and benign polyps apart from 3D colonoscopy images. The authors introduced two handcrafted convolutional neural networks called SEG and noSEG. The SEG and NoSeg networks consist of 50 3D convolutional layers, and both are stacked, forming an ensemble-based model. Both SEG and NoSEG are trained differently. On the one hand, the SEG network is trained on colonoscopy images with a mask to detect the location of polyps.

On the other hand, the NoSEG network was trained on 3D CT colonography images without masks. Both networks were trained separately to predict the class of a polyp (i.e., premalignant or benign). The output of both SEG and noSEG are concatenated, and a final class output is produced. The training dataset consisted of several privately collected images of adults undergoing colonography and cross-validated on a public dataset from the Cancer Imaging Archive (TCIA). The authors used the area under the ROC curve (AUC) and reported a score of 83.0 for the SEG network and 75.0 for the NoSEG network.

In [91], the authors introduced a polyp characterization deep learning algorithm and embedded it into a GI Genius V2 endoscopy [92]. The classification module of the proposed system consists of two pre-trained ResNet18 networks. The first network is responsible for classifying each frame as adenoma or non-adenoma. The second network produces a polyp descriptor for every detected polyp in a frame. The authors used a privately collected dataset of unfiltered colonoscopy videos for training. The same dataset was used to test the system, and an accuracy of 84.8% was reported. In addition, the authors reported a sensitivity score of 80.7% and a specificity score of 87.3%. The main limitation observed by the authors is that the model cannot classify other polyps that are neither adenoma nor non-adenoma.

A method that utilizes two pre-trained models, namely VGG16 and MobileNet, to detect polyps from colonoscopy images was presented by [93]. The authors pre-process the input images by removing the black regions in colonoscopy images, as those regions do not contain any useful information. The second pre-processing step is normalizing the RGB values to match the RGB mean of samples in the ImageNet dataset. The last pre-processing step is resizing all the input images to a constant size of 224 × 224 pixels. The images are then processed using the multi-resolution sliding window, which locates a polyp in the image. Once a polyp has been detected, the region is cropped and fed to a probability prediction function which outputs whether a polyp exists. The authors used the Kvasir-SEG dataset for training and CVC-ClinicDB and ETIS-Larib datasets for cross-validation. The precision, recall and F1 scores on the CVC-ClinciDB were 91.9, 89.0 and 0.90, respectively. When the model was tested on the ETIS-Larib dataset, a precision of 87.0, recall of 91.0 and an F1 score of 89.0 were reported.

In [16], the authors pre-trained a YOLOv3 network to detect polyps in real time. The YOLOv3 was initially pre-trained on the PASCAL VOC dataset, but later fine-tuned on 28,576 colonoscopy images. After pre-training, the authors replaced all the classes in the YOLOv3 network with one class to detect polyps, but the weights of the aeroplanes class from the PASCAL VOC dataset were reused, as they provided the best performance. Once a polyp is detected, a candidate bounding box (CBB) is integrated into the network, and it is used to filter out the network output. The CBB algorithm runs on every frame, and a threshold is set using the average of the max IoU value of the previous frame. The training dataset is private and filtered by removing all blurry images from the training and testing sets. The authors reported a precision of 89.0, recall of 87.0 and F1 score of 88.0. The authors reported that the model failed to detect flat polyps due to insufficient samples.

In [94], the authors resorted to using a classic machine learning algorithm, random forest, to detect premalignant colorectal polyps. The proposed method begins by first manually labeling the samples. At this stage, experienced colonoscopists label the samples by drawing a bounding box around a polyp, and samples with polyps that were hard to identify are excluded. The training samples are then pre-processed using an open-source Python package called Pyradiomics. The package extracts the gray-level histogram stats and image texture from the segmented photos using 22 image filters which are then used as training features. The features are fed to a random forest algorithm, and a prediction of whether a polyp is premalignant is made. Training and testing were performed using a privately collected dataset, and AUC, sensitivity and specificity scores of 91.0, 82.0 and 85.0 were reported.

Yolov5m was combined with the SWIN transformer in a study conducted by [95] to detect polyps from real-time colonoscopy videos. The authors used the YOLOv5m backbone to extract features from a single frame and combine the extracted information with the features extracted by the SWIN transformer. The authors found that combining the SWIN transformer with a local feature extraction network improves the overall performance, since each network can extract all the critical information in different spaces. The combination of the transformer and YOLOv5m network is achieved by replacing the bottleneck module of the YOLO network with the SWIN transformer. A temporal information fusion module is introduced to minimize the effect of white light reflection in each frame. The authors used the CVC-Clinic, ETIS-Larib and CVC-ClinicVideo datasets to train and test the proposed method. The model performs best when tested on the CVC-ClinicVideo, scoring a precision of 83.6, recall of 73.1 and F1 of 78.0.

An automatic polyp segmentation system was presented in [96]. The proposed method uses the SegNet architecture to segment colonoscopy images. Before training, samples are first pre-processed using a pre-defined threshold in which every image pixel is set to 50 and 15 for every red and green channel. The authors use this threshold to filter out all pixels with values below the pre-defined threshold, removing all black and non-polyp regions. The images are then fed to a SegNet model, and a polyp is localized through segmentation. The authors used CVC-Clinic, CVC-Colon and ETIS-Larib datasets to train and test the proposed model, achieving an IoU of 81.7%.

A YOLOv4-based method to detect polyps in real time was proposed in [97]. The authors pre-trained the YOLOv4 network on the ImageNet dataset, which was further improved by using the TensorRT tool to extract better performance from GPUs. The samples were resized and then augmented before feeding them to the YOLOv4 network for further processing. The authors used the Kvasir-SEG, CVC-Clinic, CVC-Colon and ETIS-Larib to train and test the proposed method. The evaluation on the ETIS-Larib produced the best performance, with a precision score of 80.53, recall of 73.56 and F1 of 76.88.

In [98], the authors introduced a plug-in module that could be integrated with any object detection algorithm. The proposed module was named instance tracking head (ITH). ITH introduces a feature-based mechanism to any detection framework to achieve a multi-task training objective. The ITH and the head of any detection algorithm share the same backbone to extract local features, which are then used for model training. The proposed plug-in was tested with the YOLOv4 algorithm, and the training dataset combined CVC-ClinicDB and CVC-VideoClinicDB. For evaluation, the authors used the ETIS-Larib dataset and reported a precision of 92.60, recall of 80.70, and F1 of 86.24.

The authors in [12] introduced a polyp detection method combining 2D and 3D convolutional layers to segment colonoscopy images. The proposed method segments colonoscopy images using a mixture of 2D and 3D backbones. The 2D convolutional layers extract all of the spatial representation, and it was pre-trained on colonoscopy images. In contrast, the 3D backbone was pre-trained on colonoscopy videos, and is used to provide a third temporal dimension. The 2D network is a pre-trained ResNet101 without dense layers. At first, the features are extracted using the 2D network; then, the 3D network generates a temporally coherent segmentation mask. The 3D network consists of two convolutional layers, followed by a dropout layer and a batch normalization layer. Finally, an interpolation layer is added to upsample the image. The method was trained on a private dataset and tested on both the SUN dataset and Kvasir-SEG. The scores presented were 86.14 for sensitivity, 85.32 for specificity, 93.45 for precision, and 89.65 for F1.

## 9. Discussion and Recommendations

Based on the reviewed literature, we can conclude that there is a specific trend among the proposed methods. First, the YOLO algorithm seems to be the most widely used method for detecting polyps in static images and from live-stream colonoscopy videos. YOLO’s popularity among researchers could be due to its ability to detect objects faster than other detection algorithms, making it the best fit for real-time polyp detection tasks. The second reason could be YOLO’s comparatively better Intersection over Union (IoU) performance in bounding boxes.

Another observation we noticed is the increased interest in using vision transformers as either a standalone network or a module integrated with another system. This trend could be because transformers are efficient compared to typical CNNs, since they divide an image into patches instead of processing the entire image pixel by pixel.

Transfer learning and pre-trained models seem to be the preferred methods of building feature extraction models or backbone networks. Transfer learning is used more frequently than CNN models built from scratch because pre-trained models are easier to fine-tune and computationally efficient. In addition, pre-trained models have been trained on larger datasets and complex tasks; therefore, one can benefit from the knowledge obtained during the initial training phase.

Based on the readings in Table 3, we observe that the average precision, recall and F1 scores are around 90.26, 86.51 and 88.38, respectively. However, studies like [11,16,98] reported scores lower than the average. In [11,16], the authors used a privately constructed dataset for training and testing that was made of samples taken in real-life scenarios without pre-processing or adjustments. In addition, [90,95] were trained and tested on a private dataset and reported the lowest specificity scores of 60.3 and 85.0, respectively. This observation indicates that even the most recent models and techniques cannot effectively handle unseen, real-life samples. This theory can be further confirmed by comparing the scores reported in [9,10]. The authors in both studies used CVC-ClinicDB as their training dataset; however, the authors in [10] reported lower scores than [9], although more training samples were used to train the model presented in [10].

The best-performing models in terms of precision, recall and F1 scores are the ones reported in [9,10,82]. The standard approach among the three methods is to utilize multiple models or paths to extract features from a given image. For example, in [9], the authors used a dual-path CNN to extract all the local and global features. Meanwhile, in [82], the authors stacked several classifiers and used the weighted prediction as the final output class. This pattern indicates that performance can be improved by combining feature maps extracted using different architectures. The observed pattern can be confirmed when examining the scores reported in [8,13], which are based on several models, versus the ones reported in [11,97], which use single sources to extract the feature map.

**Figure 4:**
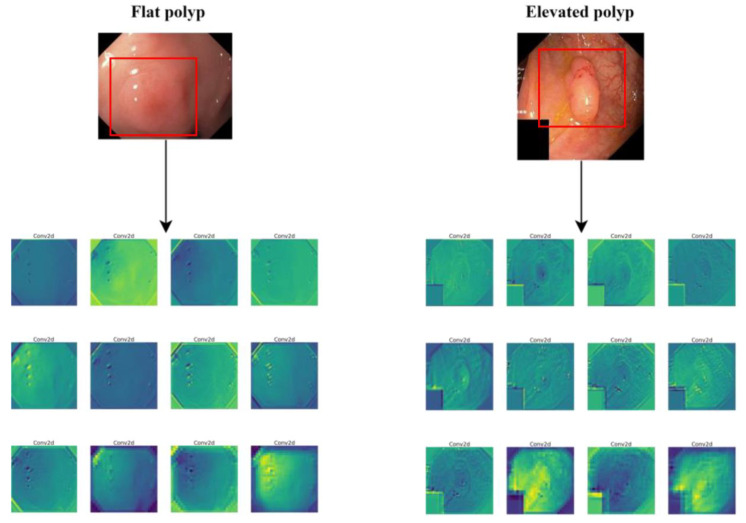
A comparison of the feature map extracted from a sample with a flat polyp (left) versus the features extract from an image with an elevated polyp. Samples were taken from Kvasir-SEG dataset.

We discovered several unaddressed gaps based on the literature reviewed in the previous section. First, many studies have reported that the many variations and sizes of polyps are one of the reasons models fail to effectively detect a polyp in a given photo or video. Usually, flat polyps that are even hard to detect with the naked eye pose the main challenge to some of the proposed models. To better visualize the challenge faced by a convolutional neural network when working on images with flat polyps, we used a pre-trained ResNet50 to extract the feature map from two samples; one has a flat polyp, and the other contains an elevated polyp. The samples were randomly picked from the Kvasir-SEG dataset; the results are presented in Figure 4.

In Figure 4, it can be observed that ResNet50 was able to detect the edges and the rounded shape of the polyp in addition to the white light reflection. In contrast, the ResNet50 network could not detect any edges or shapes when a sample with a flat polyp was provided; however, the edges of the white light reflection were extracted instead. To the best of our knowledge, there has not been an adequately tested method to optimize the extracted feature map so that flat polyps are identified, primarily from unseen samples.

The second gap is that most existing models struggle to differentiate white light reflection, fecal matter and other polyp-shaped objects from actual polyps. Despite a few studies introducing mechanisms to reduce the effect of white light reflection, such methods could not reduce the false positives caused by other factors. This issue is a potential gap for future research, since none of the methods were able to reduce the false positives caused by polyp-like objects. This issue can be observed in the features extracted in Figure 4, in which the edges of white light glare were detected and could mistakenly be identified as polyps.

Another common gap is the lack of sufficient samples to cover all the possible variations and types of polyps. The current publicly available datasets do not cover all the possible polyp variations with respect to size, shape and type. This issue is usually addressed by combining several datasets for training; however, generative adversarial networks (GANs) could augment existing samples or generate new ones.

In addition, some existing methods, like [94], discarded blurred samples that may represent real-life settings during training, causing the reported performance to be inaccurate, since it was not tested on suboptimal samples.

Besides the dataset aspect, a significant limitation identified in the current work is the gap between accuracy and optimization. Methods such as [10,12,86,89] have all produced good performances; however, because each model is complex, high-end computational resources would be a critical prerequisite for a model to run. In comparison, real-time methods such as [8,11,16,91], which attempt to detect polyps in a real-time manner, yielded comparatively lower scores. An exciting research problem would be discovering ways to optimize colorectal polyp detection models to work on low-power colonoscopies while retaining good performance.

Colonoscopes come in various resolutions and framerates, and are frequently manufactured differently, depending on the manufacturer’s engineering philosophy. To date, comprehensive tests on different colonoscopes have yet to be conducted and reported in the literature. It has been observed that the present work does not consider the specifications of the colonoscope used to collect the samples, which is a significant limitation, as one cannot confirm whether a specific model is scalable and able to work on images collected from various sources.

In future work, it would be worth looking into using vision transformers to detect and classify polyps. Additional fine-tuning of the parameters or the architecture of existing transformers might be required to fit the task of polyp detection.

Another recommended architecture that would be worth looking into is generative adversarial networks (GANs). GANs have evolved over the years, and impressive models such as StyleGAN produce realistic-looking samples, while CGAN renders samples conditionally available for the public to use. These architectures could be used to tackle the data disparity issue, either by generating new samples or augmenting existing samples during training. Aside from the technical recommendations, it is highly recommended to engage professional physicians in the industry to verify whether such methods would help reduce stress and fatigue among colonoscopists and radiologists.

## 10. Conclusions

Colorectal polyp detection using deep learning is an exciting area of research, given the advancement in the healthcare sector and the increased interest in utilizing artificial intelligence to improve efficiency and reduce the workload of physicians. However, most existing methods do not perform well in real-life settings due to several common issues. Those issues include data disparity, white light reflection from colonoscopy, demand for high-end computational resources, and the different variations, sizes and shapes of polyps. This article reviews the latest trends and methods to automatically detect colorectal polyps from colonoscopy using deep learning techniques. Our review presents the benchmark colonoscopy datasets, evaluation metrics, common challenges, and various approaches to building colorectal polyp detectors. In addition, we analyze and discuss several recently proposed methods by looking into their implementation, reported performance and limitations. We conclude the study by discussing the trends, gaps and potential future directions based on the analyzed literature.

## Figures and Tables

**Table 1:** The various classes of colorectal polyps.

Types	Average Size	Cancerous	Frequency in Patients
Tubular adenomas	1 to 12 mm	Non-cancerous	50%
Villous adenomas	≥20 mm	Cancerous	5%
Tubulovillous adenomas	≤1 cm	Cancerous	8–16%
Serrated adenomas	≤10 mm	Non-cancerous	15–20%
Hyperplastic	≤5 mm	Non-cancerous	20–40%
Inflammatory	≤2 cm	Non-cancerous	10–20%

**Table 2:** A summary of the existing benchmark colonoscopy datasets.

Name	Number of Samples	Resolution	Media Type
Kvasir-SEG	1000	Between 332 × 487 and 1920 × 1072	Static images
ETIS-Larib	196	1225 × 996	Static images
CVC-ClinicDB	612	384 × 288	Static images
CVC-PolypHD	56	1920 × 1080	Static images
CVC-ColonDB	300	574 × 500	Static images
EndoTect	110,709 images + 373 videos	-	Static images/videos
BKAI-IGH NeoPolyp-Small	1200	-	Static images
NeoPolyp	7500	-	Static images
PolypGen	8037	-	Static images
EndoScene	912	384 × 288 (CVC-ClinicDB) 574 × 500 (CVC-ColonDB)	Static images
SUN	49,136	-	Video
EndoTest	48	-	Video
ASU-Mayo clinic colonoscopy	38	-	Video
Colonoscopic Dataset	76	768 × 576	Video
CVC-ClinicVideoDB	18	768 × 576	Video

**Table 3:** A summary of all the reviewed methods.

Method	Precision	Recall	F1	IoU	Accuracy	Sens.	Spec	AUC	Training Set	Testing Set
[8]	90.61	91.04	90.82	-	-	-	-	-	SUN, PICCOLO Widefield	ETIS -Larib
[13]	92.30	-	92.40	-	-	-	-	-	EndoScene, Kvasir-SEG	EndoScene, Kvasir-SEG
[10]	94.9	96.9	95.9	-	-	-	-	-	CVC-ClinicDB, ETIS-Larib, Kvasir-SEG	Private dataset
[9]	100	99.20	99.60	-	-	-	-	-	CVC-ClincDB	CVC-ColonDB, ETIS-Larib
[14]	87.4	84.4	85.9	-	-	-	-	-	ASU-Mayo	ASU-Mayo
[58]	-	-	-	-	92%	-	-	-	University of Leeds dataset	University of Leeds dataset
[82]	98.6	98.01	-	-	-	-	-	-	Private dataset, Kvasir-SEG	Private dataset, Kvasir-SEG
[11]	78.5	78.8	78.6	-	-	-	-	-	Private dataset	Private dataset
[86]	-	-	88.0	84.2	-	-	-	-	Kvasir-SEG, CVC-ClinicDB	CVC-ColonDB, ETIS-Larib, EndoScene
[89]	-	-	97.4	-	-	97.4	60.3	91.7	Private dataset	Private dataset
[90]	-	-	-	-	-	-	-	83.0	Cancer Imaging Archive	Cancer Imaging Archive
[91]	-	-	-	-	84.8%	80.7	87.3	-	Private dataset	Private dataset
[93]	91.9	89.0	90.0	-	-	-	-	-	Kvasir-SEG	CVC-ClinicDB, ETIS-Larib
[16]	89.0	87.0	88.0	-	-	-	-	-	Private dataset	Private dataset
[94]	-	-	-	-	-	82.0	85.0	91.0	Private dataset	Private dataset
[95]	83.6	73.1	78.0	-	-	-	-	-	CVC-ClinicDB, ETIS-Larib, CVC-ClinicVideoDB	CVC-Clinic, ETIS-Larib, CVC-ClinicVideo
[96]	-	-	-	81.7	-	-	-	-	CVC-ClinicDB, CVC-ColonDB, ETIS-Larib	CVC-Clinic, CVC-ColonDB, ETIS-Larib
[97]	80.53	73.56	76.88	-	-	-	-	-	Kvasir-SEG, CVC-ClinicDB, CVC-ColonDB	Kvasir-SEG, CVC-Clinic, CVC-ColonDB
[98]	92.60	80.70	86.24	-	-	-	-	-	CVC-ClinicDB, CVC-VideoClinicDB	ETIS-Larib
[12]	93.45	-	89.65	-	-	86.14	85.32	-	SUN, Kvasir-SEG	SUN, Kvasir-SEG

## Data Availability

Not applicable.
